# Exploring Litter Decomposition, Nutrient Retention, and Sensitivity to Nitrogen Deposition Among Ancient and Recently Evolved Tree Species

**DOI:** 10.1002/ece3.71317

**Published:** 2025-04-19

**Authors:** Chaozhi Peng, Tong Chen, Wei He, Li Mei, Zeyao Zhao, Jie Fan

**Affiliations:** ^1^ College of Horticulture and Forestry Sciences/Hubei Engineering Technology Research Center for Forestry Information Huazhong Agricultural University Wuhan China; ^2^ The Forestry Prospect & Design Institute of Hubei Province Wuhan China

**Keywords:** litter decomposition, N‐addition treatment, nutrient release, plant functional types

## Abstract

Investigating the differences among plant functional types (PFTs) and their responses to N deposition is crucial for predicting carbon and nutrient cycles and improving forest management strategies. Our research aimed to examine the decomposition rates and nutrient loss rates of leaf litter and fine roots from ancient and recently evolved species and their response to N deposition. We hypothesized that (1) leaves and fine roots of recently evolved tree species decomposes slower than those of ancient tree species due to their higher C:N ratios and structural compound content; (2) the effect of N addition on decomposition rates differs across different decomposition stages and is influenced by the associated PFT; and (3) litter morphology and substrate quality are key predictors of litter decomposition rates for both ancient and recently evolved species. Field decomposition experiments were conducted with leaf litter and fine roots under both control and N‐addition treatment (10 g·m^−2^·a^−1^), focusing on three ancient tree species and three recently evolved tree species. The decomposition rate constants (*k* values) of leaves from recently evolved species were lower than those from ancient species, with values of 1.01 and 1.68 under control conditions, and 1.07 and 1.08 under N addition. For fine roots, recently evolved species had lower k values only under N addition (1.05 and 1.40), whereas no significant differences were observed between ancient and recently evolved species under control conditions. Furthermore, the N residual rate in fine roots of recently evolved species was higher under N addition compared to controls, while no such differences were observed in ancient species. The distinct patterns observed in this study provide valuable insights into the complexity of litter decomposition under N deposition, highlighting the importance of considering both PFTs and organ types for predicting ecosystem responses.

## Graphical abstract

The fine roots of evolutionary tree species have a high C:N ratio and decompose slower than those of ancient tree species. Nitrogen addition affects the decomposition of litter, and it depends on the amount of nitrogen supplied and the decomposition process. To predict the decomposition rate of litter in response to N deposition, both plant functional types (PFTs) and organ types should be considered comprehensively.

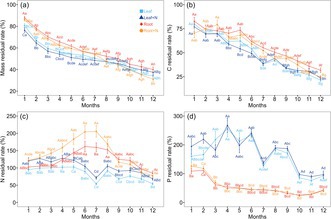

## 1 Introduction

Litter decomposition is a critical process in carbon turnover and nutrient cycling dynamics (Yang et al. 2022; Liu et al. 2023). It serves as a vital bridge connecting above‐ground vegetation to below‐ground soil, facilitating crucial plant–soil interactions (Röderstein et al. 2005). Even subtle changes in litter decomposition have the potential to significantly influence on terrestrial ecosystem C sequestration (Fan et al. 2023). Consequently, these changes can alter soil respiration (Wu et al. 2014). Current research illuminates the intricate connections between litter decomposition and nutrient release, influenced by both litter quality and environmental factors like temperature, moisture, altitude, soil properties, and soil microbial community structure (Aerts 1997; Cornwell et al. 2008; Preston et al. 2009; Joly et al. 2023). Litter with higher quality (such as higher N and P concentrations, lower C:N and lignin:N ratios) tends to have a faster decomposition rate (higher *k* values), mainly because high‐quality litter is more easily utilized by microorganisms (Prescott 2010; Liu et al. 2024). Variations in these factors significantly modulate *k* values, thereby impacting the efficiency of carbon and nutrient cycling in ecosystems. Additionally, litter morphology, including traits such as specific leaf area (SLA) and specific root length (SRL), can significantly influence the decomposition rate by affecting the surface area available for decomposers and altering the accessibility and efficiency of microbial breakdown (Makita and Fujii 2015).

Global changes significantly influence litter decomposition by modulating both the internal and external environments of the decomposition process, including factors such as initial litter quality and soil properties (Austin and Vitousek 2000; Hou et al. 2021). Decomposition rates are commonly quantified using the *k* values, which represent the rate at which litter decomposes over time. Numerous studies have examined how global change factors (GCFs) affect *k* values across various ecosystems, revealing complex interactions between environmental factors and litter quality (Zhang et al. 2018; Su et al. 2023; Wu et al. 2023). Nutrient addition, specifically, exerts a dualistic effect on litter decomposition. On the one hand, the improvement in litter quality resulting from nutrient addition, such as increased availability of nitrogen (N) and phosphorus (P), stimulates microbial activity, thereby accelerating the decomposition process (Zhu et al. 2016; Hou et al. 2021; Song et al. 2016; Wu et al. 2023). On the other hand, nutrient addition may also adversely affect soil microbial activity by lowering soil pH, thereby slowing down the decomposition process (Zhang et al. 2018). The net impact of nutrient addition on litter decomposition ultimately depends on the delicate equilibrium between its inhibitory and stimulatory mechanisms, as noted by Liu et al. (2024).

Despite significant progress in understanding the processes of litter decomposition, as well as its response to GCFs, there remains a gap in our knowledge regarding the decomposition patterns of various plant functional types (PFTs). N deposition, as an important global change factor, exerts direct and indirect influences on litter decomposition by altering soil N availability, microbial activity, and community (Britton et al. 2018). Consequently, advancing knowledge in this field is essential for accurately predicting forest ecosystem responses and developing effective management strategies in the context of global change.

Plant functional types classified based on morphological, physiological, and life‐history attributes that confer comparable ecosystem impacts (Duckworth et al. 2000) exhibit marked differences in litter decomposition rates, attributable to their inherent chemical and physical characteristics (da Silva et al. 2022; Hobbie 2015). For instance, litter of coniferous species decomposes more slowly than that of deciduous and evergreen broad‐leaf species due to their elevated lignin/N and C/N ratios (Zhang et al. 2019). Conversely, N‐fixing species accelerate litter decomposition by enhancing soil N content through biological N‐fixation, resulting in higher N levels and lower lignin content in their litter (Wedderburn and Carter 1999). Concurrently, tree species originating from the late Cretaceous era onwards exhibit a discernible pattern of primary root diameters initially declining and subsequently stabilizing (Chen et al. 2013). This phenomenon may mirror the transition towards more arid conditions and localized habitat alterations since the mid‐Cretaceous epoch, potentially associated with global climate shifts occurring 120 million years ago (Crepet et al. 2004). Angiosperms that have evolved since then exhibit not only diminished root diameters but also thinner cortex and a reduction in cortical cell layers (Gu et al. 2014), adaptations that improve their capacity for efficient water and nutrient uptake (Comas et al. 2012). Given these evolutionary trends and anatomical adjustments, we hypothesize that ancient and recently evolved species might exhibit divergent decomposition rates for their roots and leaf litter. Nevertheless, no studies have directly compared the decomposition dynamics between roots and leaf litter across these two taxonomic categories, emphasizing the need for further exploration. Furthermore, if ancient and recently evolved tree species, categorized by distinct PFTs, exhibit varying decomposition rates for their above‐ and below‐ground litter, it is imperative to identify the key factors governing these differences. Thus, exploring the decomposition patterns and identifying the critical determinants influencing decomposition between ancient and recently evolved species is vital to enhance comprehension in this area. This effort requires clarifying the dynamic mechanisms through which global change, particularly N deposition, impacts litter decomposition across different PFTs, and how these alterations subsequently resonate through carbon and nutrient cycling and ecosystem services within forest ecosystems. These understandings will provide a solid scientific basis for developing forest management strategies that can respond to global change.

N deposition in forest ecosystems has multiplied due to fertilization and fossil fuel combustion, and it is expected to increase continuously in the future (Yu et al. 2019). The elevated atmospheric N deposition, coupled with human‐induced N inputs, has augmented N availability in forest soils, ultimately modifying soil fertility, litter quality, and microbial communities, thereby exerting a profound influence on litter decomposition dynamics within forest ecosystems (Morrison et al. 2018). While extensive research has explored the effects of N deposition on litter decomposition, results often diverge due to the intricate interplay of diverse influencing factors (Knorr et al. 2005; Zhang et al. 2018; Su et al. 2021). Specifically, N deposition alters soil environments and the initial chemical composition of litter, thereby regulating litter decomposition (Britton et al. 2018; Ding et al. 2022). Given their distinct litter morphology and material composition, ancient and recently evolved species may exhibit varying responses to N deposition during litter decomposition. Consequently, it is imperative to delve into the differences in litter decomposition among different functional tree species under anticipated N deposition scenarios.

We conducted a year‐long litter decomposition experiment, featuring three ancient tree species and three recently evolved species. By simulating N deposition through the application of N fertilizer, we examined the decomposition patterns of fine roots and leaves litter in both ancient and recently evolved species. We hypothesized that (1) Leaves and fine roots of recently evolved tree species decompose slower than those of ancient tree species due to their higher C:N ratios and structural compound content; (2) The effect of N addition on decomposition rates differs across different decomposition stages and is influenced by the associated PFT; and (3) Litter substrate traits are key predictors of litter decomposition rates for both ancient and recently evolved species. Our objective is to identify differences in litter decomposition characteristics and their response to nitrogen deposition between ancient and recently evolved tree species, as well as the key factors influencing this process.

## 2 Materials and Methods

### 2.1 Study Sites

The research was conducted in Taizi Mountain, Jingshan County, Hubei Province, China (112°48′45″ ~ 113°03′45″ E, 30°48′30″ ~ 31°02′30″ N), a region characterized by low‐lying hills and a yellow‐brown soil. The region is classified as semi‐tropical, exhibiting a humid monsoon climate. The seasons are distinguished by contrasting precipitation patterns: winter and spring are dry, while summer and autumn are characterized by high levels of rainfall. The region experiences an average annual rainfall of 1094.6 mm, with an average annual temperature of 16.4°C. The lowest monthly average temperature is recorded in January at 2.6°C–3.0°C, while the highest is observed in July at 28.8°C. The region has 240 days per year free of frost.

### 2.2 Experimental Design

In this study, six tree species were selected and classified based on the APG IV plant classification system (Chase et al. 2016), which groups plants according to their evolutionary relationships. Using the Phylomatic and Phylocom software along with the Bladj age algorithm, we estimated the evolutionary divergence times of these species. Based on this information, the species were categorized into two PFTs. The first category, “ancient species,” comprised *Liriodendron chinensis*, *Eucommia ulmoides,* and *Liquidambar formosan*a. The second category, “recently evolved species,” included *Ilex purpurea*, *Paulownia tomentosa,* and *Quercus acutissima* . Furthermore, the provenance of these six species can be referenced from a previous study (Wikström et al. 2001) (Table S1). In July 2020, we established four replicate plots (10 m ×15 m of each plot) for each species. Two 2 m × 3 m subplots were established within each replicate plot, with a 3 m buffer zone between them, to simulate N deposition with and without N‐addition treatment (control). A total of 48 plots were established. To simulate nitrogen deposition, urea (CO(NH_2_)_2_) was applied to the 24 N‐fertilized plots, with a total N amount of 10 g N·m^−2^·a^−1^ (+N). From August 2020 until August 2021, urea was applied every 3 months. The selection of urea was primarily based on its status as a principal constituent of organic N in atmospheric N deposition (Cornell 2011).

### 2.3 Decomposition and Sampling

In August 2020, a sample of freshly fallen leaves was collected using specially designed collection nets. Subsequently, all leaf samples were subjected to a drying process until they reached a constant mass. Concurrently, fresh roots of the six tree species were harvested with a shovel from the upper 20 cm of soil in plots that had not been treated. All root samples were subjected to a washing process with the objective of removing soil particles and subsequently air‐dried until reaching a constant mass over a period of 14 days. Subsequently, fine roots (diameter < 2 mm) were selected for the decomposition study.

Prior to the placement of leaves and roots materials in the litter bags, all samples within each species were thoroughly mixed to minimize variation between litter bags. Subsequently, 10.0 g of leaf samples and 5.0 g of absorbent roots were placed in nylon bags (15 cm × 15 cm, with 0.05 mm mesh size). On 25 August 2020, 12 leaf litter bags were laid flat on the ground, and 12 root litter bags (comprising 6 tree species, 2 treatments, 4 replicate plots, and 12 sample times per year) were placed vertically at a depth of 0–15 cm in each plot of each tree species. The bags were separated by approximately 15 cm, secured with wire, and labeled. Samples were taken monthly over the course of the 1‐year decomposition period, beginning in September 2020 and concluding in September 2021. A total of 576 leaf litter bags and 576 root litter bags were prepared for the decomposition experiment, with 6 species, 2 treatments, 4 replicate plots, and 12 samples per year. A total of 96 bags were collected for each treatment and species, with four replicate bags for each litter type (leaves and roots) and two treatments, across four replicate plots. The litter bags were transported to the laboratory for further analysis. The roots were meticulously cleaned to eliminate mineral soil particles and debris, then subjected to a 72‐h drying process at 65°C. Following this, the residual mass was determined through weighing. Subsequently, the litter was powdered and passed through a 0.15 mm sieve. The sieved litter samples were subjected to chemical analysis.

### 2.4 Leaf and Fine Root Analysis

The C content of the leaves and fine roots litter was determined through the application of the K_2_Cr_2_O_2_ oxidation method. The total N and total P in the leaves and fine roots litter were analyzed using an automatic discontinuous element analyser (Autochem1100 & 1200) following digestion in a solution of concentrated sulfuric acid (98%) and perchloric acid. The cellulose and lignin contents were determined by an improved acid wash method, as described by Vanderbilt et al. (2008). The leaf and fine root morphological traits were determined based on the methods reported by Cornelissen et al. (2003). The fresh fine roots were scanned with an Epson scanner (12,000 XL), and the root morphology parameters such as root diameter, length, root surface area, and root volume were analyzed by the WinRHIZO analysis system software (Pro 32‐bit 2019a). Leaf area was analyzed using a Leaf Area Analyzer (LA‐S, China). Then the scanned roots and leaves were oven‐dried at 65°C until constant weight to calculate the specific root length and specific leaf area.

### 2.5 Calculations and Statistical Analyses

The mass residual rate (*W*) and the nutrient residual rates (N) were expressed as follows:

(1) $$W=M_{t}/M_{0}\times 100$$

(2) $$N=C_{t}M_{t}/C_{0}M_{0}\times 100$$

where *C*_*0*_ is the initial nutrient concentration of the leaves and fine roots, *C*_*t*_ is the nutrient concentration of the leaves and fine roots at time *t*, *M*_*t*_ is the remaining litter mass after a given time period *t* (g), and *M*_*0*_ is the initial litter mass (g).

Decomposition constants (*k*) of the litter were calculated according to the following equation (Olson 1963):

(3) $$M_{t}/M_{0}=e^{-\mathrm{kt}}$$

where *k* is the decomposition rate constant, and *t* is the decomposition time. The model was fitted using data from 12 time points across the decomposition period, ensuring that *k* accurately reflects the overall decomposition dynamics. To assess the effects of PFT (nested within species), N‐addition treatment, decomposition time, and their interactions on the residual rates of mass, C, N, and P, we used repeated‐measures ANOVA via the “lmer” function from the lmerTest package. Two‐way ANOVA was employed to examine the effects of N‐addition treatment and PFT along with their interaction on the litter *k* values. Turkey's HSD test was utilized for multiple comparisons of N‐addition treatment, time, and PFT. Principal component analysis (PCA) was executed using the stats package and the factoextra package. Paired t‐tests were conducted to analyze differences in initial traits between leaves and fine roots, with a significance threshold set at *p* < 0.05. Independent samples t‐tests were also carried out to assess the differences in initial traits of leaves and fine roots across various PFTs, maintaining the significance level at *p* < 0.05. To determine the most effective initial trait model for predicting *k* values in leaves and fine roots, various combinations of predictor variables were analyzed using general linear models (GLMs) to evaluate the extent to which morphological and chemical indicators can forecast the *k* value. Pearson correlation analysis was used to test for significant correlations between initial traits and the k value. The variables identified as significant predictors were used only when they showed a correlation with *k* values. To address multicollinearity among the predictor variables, we removed one of the two predictors that had a correlation coefficient of *r* > 0.7 from the model, following the guidelines set by Dormann et al. (2013). A collection of models featuring combinations of predictors were constructed in the global model through the MuMIn package (Barton 2009). Models with the best AIC scores (ΔAIC ≤ 2) were chosen. The explained variance for each of the leading models was assessed using the rsq package. All statistical analyses were performed in R (v4.3.3, R Core Team 2019).

## 3 Results

### 3.1 Trait Variations in Leaves and Fine Roots Across Different PFTs

**FIGURE 1:**
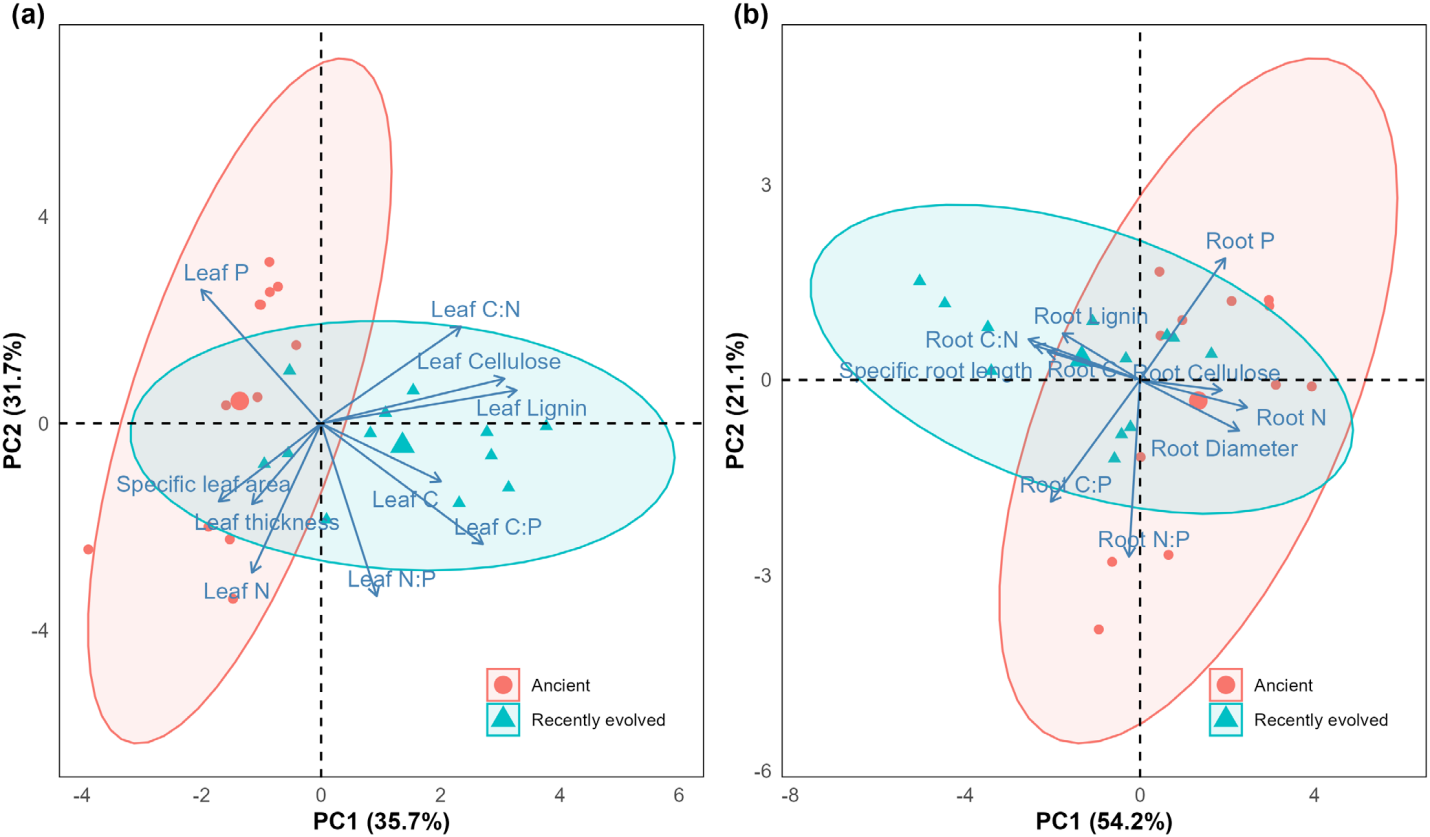
Biplots of the principal component analysis (PCA) of morphological and chemical traits of leaves (a) and fine roots (b) among plant functional types (PFTs).

The PCA ordination analysis of leaves traits indicated that the first axis, accounting for 35.7% of the total variation, effectively distinguished between PFTs with statistical significance (F [1, 22] = 26.06, *p* < 0.001; Table S2). In contrast, the second axis, explaining 31.7% of the total variation, failed to separate PFTs, as evidenced by non‐significant results (F [1, 22] = 1.439, *p* = 0.243; Table S2). When comparing recently evolved species to ancient species, the former exhibited higher C:N ratios, lignin, and cellulose contents in their leaves (Figure 1; Table S3). Similarly, in the PCA of fine root traits, the first axis, which accounted for 54.2% of the total variation, significantly differentiated between PFTs (F [1, 22] = 11.47, *p* < 0.01; Table S2). Conversely, the second axis, explaining 21.1% of the variation, did not show a significant separation among PFTs (F [1, 22] = 1.169, *p* = 0.291; Table S2). In terms of fine root characteristics, ancient species had higher N content, cellulose, and diameter, whereas recently evolved species possessed higher C:N ratios and specific root lengths (Figure 1; Table S3).

A comparative analysis revealed that leaves tissues had significantly higher C and N contents, C:P and N:P ratios, and cellulose levels compared to fine roots. Conversely, leaves exhibited lower P content and C:N ratios when compared to fine roots (Table S4).

### 3.2 Effects of N Deposition, PFT, and Decomposition Time on Litter Mass Residual Rate

The repeated‐measures ANOVA analysis revealed that both N‐addition and decomposition time, along with their combined two‐way and three‐way interactions with PFT, had significant impacts on the residual mass of leaf litter (Table 1). Notably, PFT alone did not exhibit a direct effect on leaf litter's residual mass. In contrast, for fine roots, the residual mass was influenced by N addition, decomposition time, and their interactive effects (Table 1).

**TABLE 1:** Results of repeated‐measures ANOVA: Effects of N‐addition treatment (Na), decomposition time (T), PFTs (nested within species), and their interactions on residual rates of mass, C, N, and P in decomposing litter.

Source	Mass		C		N		P	
	*F*	*p*	*F*	*p*	*F*	*p*	*F*	*p*
Leaves								
Na	13.609	**< 0.001**	1.382	0.240	110.095	**< 0.001**	2.491	0.115
PFTs	2.929	0.162	2.790	0.170	0.085	0.785	5.624	0.077
T	818.887	**< 0.001**	239.046	**< 0.001**	37.703	**< 0.001**	30.800	**< 0.001**
Na × PFTs	11.222	**< 0.001**	0.029	0.865	2.367	0.124	1.185	0.277
Na × T	11.054	**< 0.001**	2.020	**0.025**	0.575	0.849	1.103	0.356
PFTs×T	7.303	**< 0.001**	5.477	**< 0.001**	3.338	**< 0.001**	2.704	**< 0.001**
Na × PFTs×T	2.099	**0.019**	0.799	0.642	0.7353	0.705	0.692	0.747
Roots								
Na	73.570	**< 0.001**	26.159	**< 0.001**	63.784	**< 0.001**	1.824	0.177
PFT	1.375	0.306	1.143	0.345	2.670	0.178	0.790	0.424
T	437.683	**< 0.001**	174.735	**< 0.001**	68.244	**< 0.001**	152.074	**< 0.001**
Na × PFT	0.797	0.372	0.835	0.361	16.649	**< 0.001**	1.305	0.254
Na × T	3.524	**< 0.001**	3.043	**< 0.001**	7.669	**< 0.001**	1.713	0.067
PFT × T	1.098	0.361	6.872	**< 0.001**	14.7091	**< 0.001**	9.682	**< 0.001**
Na × PFT × T	1.071	0.393	1.341	0.198	0.857	0.583	1.145	0.323

*Note:* The *p*‐values in bold indicate significant terms at *p* < 0.05.

**FIGURE 2:**
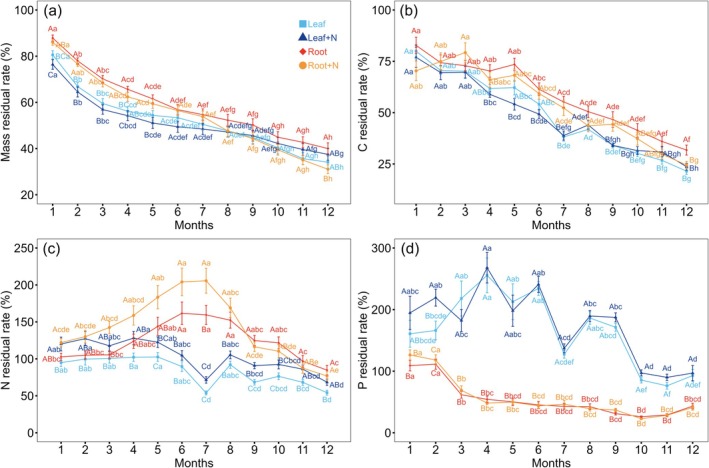
Comparison of residual rates of litter mass (a), carbon (b), N (c), and P (d) (mean ± S.E.) across different decomposition times and litter treatments. The same lowercase denotes non‐significant difference among different decomposition times for each of litter treatments and the same uppercase denotes non‐significant difference among litter treatments for each of decomposition times at *p* < 0.05. Root+N and Leaf+N represent N‐addition treatments applied to root and leaf litter.

**FIGURE 3:**
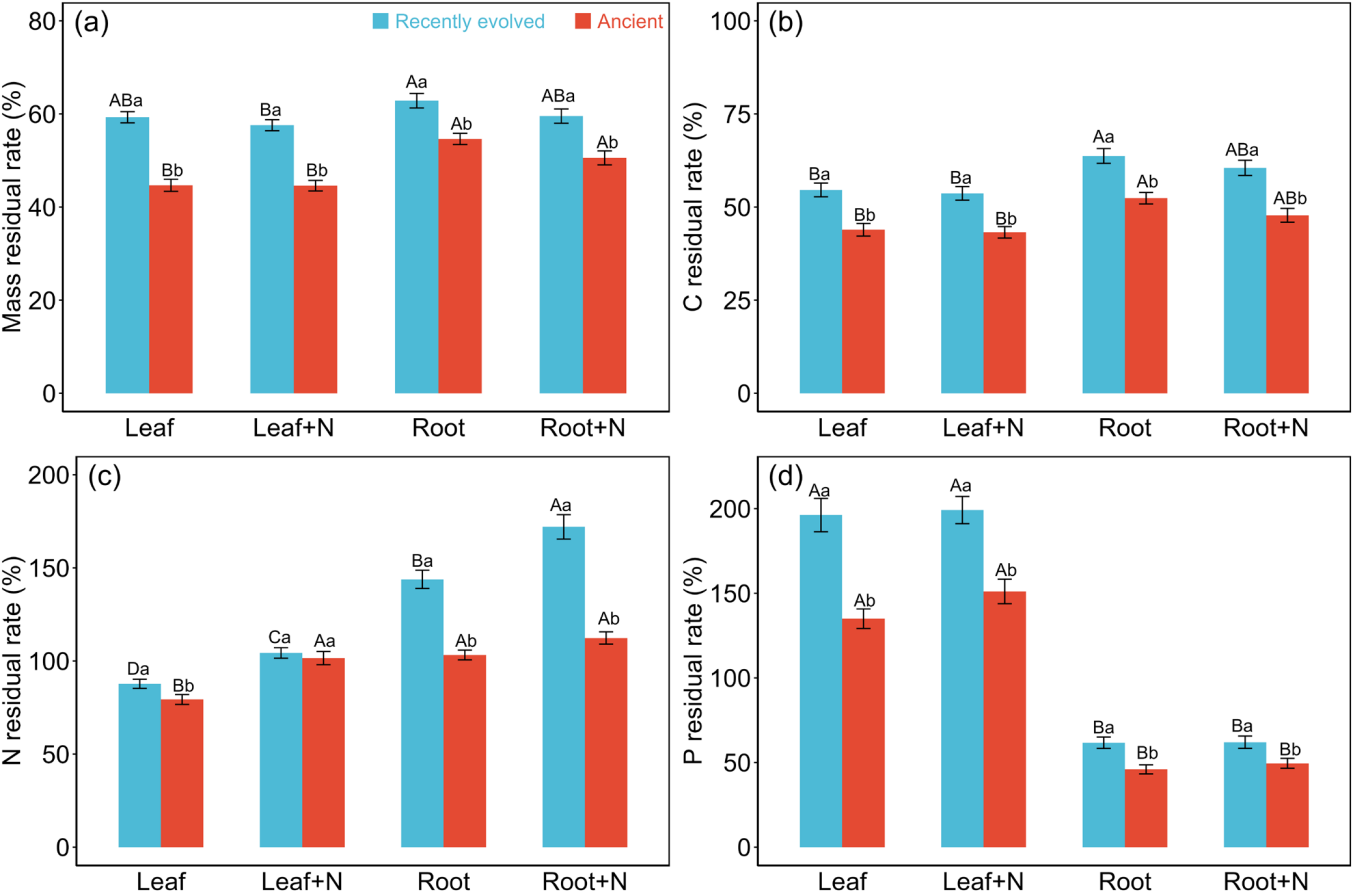
Residual rates of litter mass (a), carbon (b), nitrogen (c) and phosphorus (d) (mean ± S.E.) among plant functional types (PFTs) for different treatments. The same lowercase denotes non‐significant difference among different PFTs for each of treatments and the same uppercase denotes non‐significant difference among treatments for each of PFTs at *p* < 0.05. Root+N and Leaf+N represent N‐addition treatments applied to root and leaf litter.

Across the entire decomposition process, a similar trend was observed in the residual mass patterns of both leaves and fine roots across different N treatments (Figure 2a). After one year, the residual mass of leaf litter in the control (34.10% ± 2.64%) and N‐fertilized groups (37.52% ± 2.58%) showed no significant difference, mirroring the lack of a significant difference between control and N‐fertilized fine roots at this point. However, for fine roots specifically, the control group (40.00% ± 2.47%) had significantly higher residual mass compared to the N‐fertilized group (31.05% ± 1.88%) (Figure 2a). Furthermore, an analysis of recently evolved versus ancient species revealed distinct patterns. Throughout the decomposition, leaves from recently evolved species consistently retained more mass than those from ancient species, as indicated by a significant PFT × T interaction (*p* < 0.001; Figure S1a). For fine roots, this trend was observed only during the first six months of decomposition, with no significant difference between recently evolved and ancient species thereafter (PFT × T, *p* < 0.001; Figure S1a). Lastly, under different N‐ addition treatments, the residual mass of leaf litter from recently evolved species was significantly greater than that of ancient species, demonstrating a significant *N* × PFT interaction (*p* < 0.001; Figure 3a).

### 3.3 Effects of N Deposition, PFTs, and Decomposition Time on Litter Nutrient Residual Rate

The residual rates of C, N, and P in leaf litter and fine roots were intricately influenced by various factors. Notably, the residual rates in leaf litter were modulated by decomposition time and the interaction between PFT and time (Table 1). Additionally, C residual rates in leaf litter were further influenced by the joint effect of N‐ addition and decomposition time, whereas N residual rates were solely impacted by N‐ addition (Table 1). In fine roots, C and N residual rates were shaped by N‐ addition, decomposition time, and their interactions, along with the interaction between PFT and decomposition time. Notably, N residual rates in fine roots were also influenced by the interaction between N‐ addition and PFT. P residual rates, on the other hand, were primarily driven by decomposition time and the interaction between time and PFTs (Table 1).

During the decomposition period, a similar pattern emerges in the C residual rates of both leaf litter and fine roots, with significant differences between control and N‐fertilized samples, particularly in the interaction between N‐ addition and decomposition time (*N* × T). Specifically, after 1 year of decomposition, N‐fertilized fine roots exhibit a lower C residual rate compared to the control (Figure 2b). Furthermore, throughout the decomposition process, recently evolved species consistently display higher residual rates of C in both leaf litter and fine roots when compared to ancient species, as evidenced by the significant interaction between PFT and decomposition time (PFT × T, *p* < 0.001) (Figure S1b). During the decomposition process, a significant divergence in N dynamics was observed between leaf litter and fine roots regarding the impact of PFT, (represented by recently evolved and ancient species). Specifically, while there was no discernible difference in N residual rates between the leaf litter of recently evolved and ancient species (PFT × T, *p* < 0.001; Figure 3c), the fine roots of recently evolved species consistently exhibited higher N residual rates compared to those of ancient species (PFT × T, *p* < 0.001; Figure 3c).

In terms of N‐addition treatments, an initial trend was observed in the first 7 months of decomposition, where N‐fertilized fine roots showed higher N residual rates than their control counterparts. However, as the decomposition progressed, this difference dissipated, resulting in no significant variation in N residual rates between N‐fertilized and control fine roots (*N* × T, *p* < 0.001; Figure 2c). This pattern was further nuanced by the PFT, as N‐fertilized fine roots from recently evolved species maintained a higher N residual rate than their control, whereas no such difference was noted in ancient species (PFT × *N*, *p* < 0.001; Figure 3c). Fine roots and leaf litter exhibited distinct nitrogen dynamics during decomposition, with fine roots showing a pattern of initial nitrogen accumulation followed by release, whereas leaf litter displayed a relatively stable nitrogen release process (Figure 2c).

Regarding P dynamics, a clear distinction emerged in leaf litter between recently evolved and ancient species throughout the 1‐year decomposition period, with recently evolved species consistently displaying higher P residual rates (Figure 3d). In contrast, no significant difference in P residual rates was observed between the fine roots of recently evolved and ancient species. Fine roots and leaf litter exhibited distinct phosphorus dynamics during decomposition, with fine roots showing a rapid decline in phosphorus residual rates during the first 3 months, followed by a relatively stable phase. In contrast, leaf litter demonstrated no clear decomposition pattern, fluctuating throughout the decomposition period but exhibiting an overall decreasing trend (Figure 2d).

### 3.4 The Effect of N‐Addition on Litter Decomposition k Values of PFTs

**FIGURE 4:**
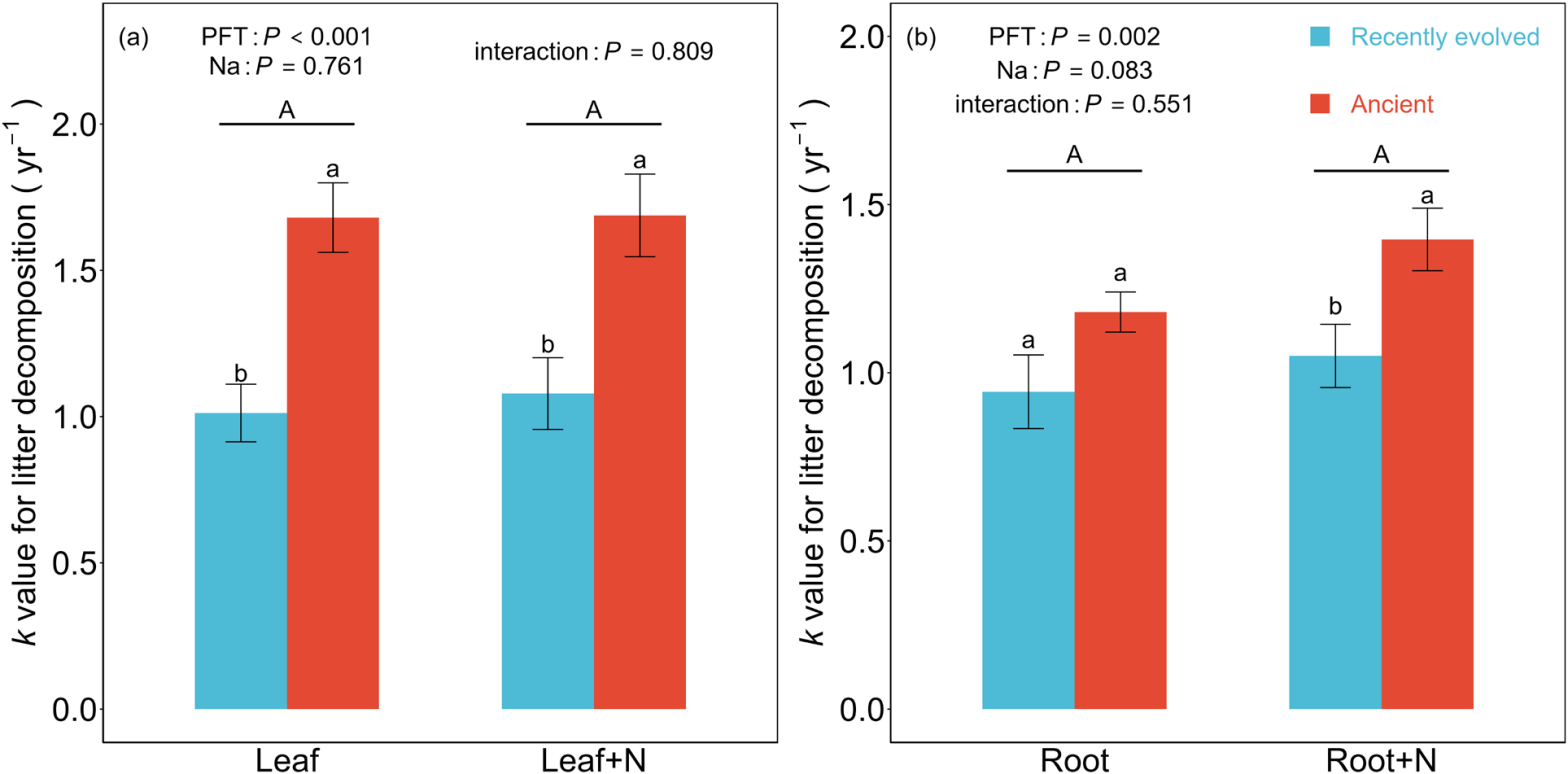
Differences in decomposition *k* values (yr^−1^) of leaves (a) and fine roots (b) of plant functional types (PFTs) under different N‐addition treatments. The same lowercase denotes non‐significant difference between PFTs for each of N‐addition treatment and the same uppercase denotes non‐significant differences between the N‐addition treatments (*p* < 0.05). Root+N and Leaf+N represent N‐addition treatments applied to root and leaf litter.

The two‐way ANOVA analysis revealed a profound influence of plant functional type (PFT) on the *k* values of both leaf litter (*p* < 0.001; Figure 4a) and fine roots (*p* = 0.002; Figure 4b). Notably, neither N‐addition nor its interaction with PFT significantly altered these *k* values for either litter type (Figure 4a,b). Intriguingly, ancient species displayed notably higher decomposition rates compared to recently evolved species for both leaf litter and fine roots (Figure 4a,b).

Model selection, guided by the criterion of ΔAIC ≤ 2, indicated that for ancient species, the *k* value of leaf litter decomposition was predominantly predicted by the leaves' C:N ratio, accounting for 90.26% of the total variability. In contrast, for N‐fertilized leaf litter from ancient species, the predictive power encompassed leaves N content and leaves thickness, with the top two models collectively explaining 97.80%–98.95% of the total variance. Regarding fine roots from ancient species, the decomposition *k* value was primarily predicted by root diameter, capturing 70.87% of the total variance. In the case of recently evolved species, fine root decomposition rates were jointly predicted by the root C:P ratio, root lignin content, root P content, and root diameter, with the top two models accounting for approximately 97% of the total variability. For N‐fertilized fine roots from recently evolved species, the key predictors were root N content, root P content, and root diameter, with the top three models explaining 92.44%–96.88% of the total variance (Table 2).

## 4 Discussion

### 4.1 Plant PFTs and Litter Decomposition Dynamics

Plant functional types (PFTs) play a key role in shaping litter decomposition processes, as evidenced by the diverse leaves and fine roots traits observed across species (Sun et al. 2022; Wright et al. 2005; Rahman and Tsukamoto 2013; Erdenebileg et al. 2022). The study conducted by Yang et al. (2022) revealed that litter quality played a more significant role in litter decomposition compared to fauna and the specific decomposition site within temperate forest ecosystems. Our study provides intriguing insights into how PFTs influence decomposition rates, demonstrating that recently evolved tree species exhibit lower decomposition k values for both leaf litter and fine roots compared to ancient tree species (Figure 4a,b). This observation aligns with Hypothesis 1, which suggests that leaves and fine roots of recently evolved tree species, due to their higher C:N ratios, decompose more slowly than ancient species. The slower decomposition rates observed in the leaves and fine roots of recently evolved species may stem from their inherently higher C:N ratios, which decelerate decomposition due to soil microorganisms' reliance on external N sources for growth (Cornwell et al. 2008; Hobbie 2005). Moreover, recently evolved species leaves are enriched in lignin and cellulose (Figure 1; Table S3), structural compounds that hinder microbial colonization and invertebrate feeding, further slowing down decomposition (Austin and Ballaré 2010; Xu et al. 2020).

In the context of global N deposition, our findings underscore the complexity of predicting litter decomposition solely based on PFTs, as the dynamics vary significantly depending on plant organs and classification criteria. This complexity supports Hypothesis 2, which posits that the effects of N addition on decomposition rates are not uniform but instead vary with time and the specific plant functional type involved. Thus, future research endeavors should delve deeper into the differential effects of various PFTs on litter decomposition, fostering a more comprehensive understanding of the intricate relationships between PFTs and ecosystem processes.

### 4.2 N Addition Effect on Litter Decomposition and Nutrient Release

The current literature on the impacts of N addition on litter decomposition remains inconclusive, with reports of positive, negative, and neutral effects (Knorr et al. 2005; Zhang et al. 2018; Gill et al. 2021; Wu et al. 2023; Wang et al. 2024). The addition of N resulted in an elevation of litter N content and a corresponding reduction in the C:N ratio, both in common site experiments and in situ conditions. This shift facilitated an increase in the *k* values (Prescott 2010; Bradford et al. 2016; Wu et al. 2023). However, in our study, no significant influences were found of N addition (at a rate of 100 kg ha^−1^ yr.^−1^) on the *k* values of both leaves and fine roots litter (Figure 4a,b). This lack of effect may stem from the moderate level of N addition employed, as meta‐analyses suggest that low N addition rates (< 50 kg ha^−1^ yr.^−1^) tend to have minimal impacts on litter decomposition rates (Wu et al. 2023). Moreover, the site‐specific environmental N deposition levels may have already mitigated N limitation, enabling microbial communities to acclimate to additional N inputs. Notably, N deposition elicits distinct responses in N release from fine roots between ancient and recently evolved species. Specifically, fine roots litter in N‐fertilized plots from recently evolved tree species show a higher N residual rate compared to controls, whereas no such difference is observed in ancient tree species (PFT × *N*, *p* < 0.001; Figure 3c). This disparity underscores the nuanced interplay between PFTs and N deposition in modulating litter decomposition.

Furthermore, our findings challenge the notion that N addition consistently constrains litter decomposition and nutrient release over time (Zhang et al. 2018). Instead, we observed that during the initial 7 months of decomposition, N‐fertilized fine roots exhibited a higher N residual rate compared to controls (*N* × T, *p* < 0.001; Figure 2c), possibly due to increased soil N availability fostering enhanced N retention by fine roots. However, this difference vanished in the subsequent 5 months, suggesting that the stimulation effect of N addition on fine root N content may be transient and dependent on the decomposition stage. This observation aligns with Hypothesis 2, which suggests that the effect of N addition is time‐dependent, promoting N retention early in the decomposition process but diminishing over time.

Throughout the decomposition process, no significant difference was observed in the C residual rates between N‐fertilized and control fine roots. However, after a year of decomposition, a notable decrease in the C residual rate was detected in N‐fertilized fine roots compared to controls. This trend could potentially be attributed to enhanced microbial activity in response to N addition over time, which may have accelerated the decomposition of the fine root litter. As microbial activity increases, the decomposition of non‐structural carbohydrates in the fine roots is likely to accelerate, leading to a reduction in C content within the fine roots (Tan et al. 2020).

### 4.3 Litter k Values Models Prediction

Our analysis highlights the strong predictive power of leaf morphological and chemical traits in explaining over 90% of the variation in decomposition rates of leaf litter from ancient species under different N‐addition treatments (Table 2). In particular, the C:N ratio emerges as the primary predictor, reflecting the essential balance between carbohydrates and proteins in the litter substrate and aligning with previous studies that emphasize its role in litter turnover rates (Talbot and Treseder 2012; Liu et al. 2018). This finding supports our Hypothesis 3, which suggests that leaf traits such as the C:N ratio and root diameter are important indicators in predicting litter decomposition rates.

**TABLE 2:** Optimal models for predicting decomposition rate constants (*k* values) of leaves and fine roots litter across N‐addition treatments and plant functional types (PFTs), based on morphological and chemical attributes (ΔAIC ≤ 2).

PFTs	Organ	Treatment	Model	df	AICc	*R* ^2^ (%)
Ancient species	Leaves	CK	3.698–0.053 leaf C:N	7	−1.36	90.26
		+N	−0.456 + 0.191 leaf N	7	−11.40	97.80
			−0.294 + 0.151 leaf *N* + 0.908 leaf thickness	6	−10.82	98.95
	Root	CK	0.580 + 0.739 root diameter	7	−4.17	70.87
Recently evolved species	Root	CK	2.255–0.001 root C:P‐0.029 root lignin	6	−7.65	97.39
			−0.965 + 1.961 root *P* + 1.447 root diameter	6	−6.92	97.18
		+N	−0.622 + 0.316 root *N* + 0.792 root P	6	−8.54	96.88
			−0.5567 + 0.414 root N	7	−7.77	92.44
			−0.603 + 1.625 root *P* + 1.332 root diameter	6	−7.38	96.45

However, when these leaf traits are applied to recently evolved species, their predictive capacity significantly diminishes, suggesting that species‐specific chemical and morphological variations within different PFTs may substantially influence decomposition modeling outcomes. In contrast to leaf traits, root diameter demonstrates a strong predictive ability for fine root decomposition, accounting for a substantial portion (70.87% ~ 97.18%) of the total variance observed (Table 2). This underscores the role of root diameter as a key morphological trait influencing fine root decomposition processes. Interestingly, specific root length (SRL), which is often considered an important predictor, fails to predict root decomposition rates in our study. This discrepancy may result from focusing on fine roots (*d* < 2 mm) rather than first‐order roots, which could obscure the direct influence of morphological traits on fine root decomposition. This result partially contradicts Hypothesis 3, indicating that the predictive power of different traits may depend on specific plant organs and types.

Overall, our findings partially validate Hypothesis 3 by highlighting the importance of the C:N ratio and root diameter as effective predictors while also identifying the limitations of SRL as a predictive trait. These results provide valuable insights into the complex interactions between litter quality, N‐addition treatments, and decomposition rates and emphasize the need to refine global carbon cycle models by incorporating species‐specific litter traits and their interactions with environmental factors such as N deposition.

## 5 Conclusion

This study explored the intricate differences in litter decomposition among different PFTS, with a particular emphasis on their responses to nitrogen (N) deposition. Our findings show that, under both control and N addition conditions, the decomposition rate constant (*d* values) of leaf litter from recently evolved species was consistently lower than that of ancient species. Regarding fine roots, no significant difference was detected between species under control conditions. Nevertheless, under N addition, the decomposition rate of fine roots from recently evolved species decelerated significantly, suggesting a greater sensitivity to N deposition. Significantly, the N residual rates in the fine roots of recently evolved species under N‐addition conditions were higher than those in control roots, while ancient species did not exhibit such differential responses.

These results highlight that litter decomposition responses to N deposition are species‐specific and organ‐specific, highlighting the complexity of these ecological interactions. However, given the relatively short 1‐year observation period, the long‐term effects of N deposition on litter decomposition dynamics remain uncertain. Longer‐term studies are needed to assess the stability of these patterns over time. By uncovering these intricate relationships, this study provides a theoretical foundation for predicting material cycles and ecological functions under N deposition for two distinct PFTs.

## Author Contributions

**Chaozhi Peng:** data curation (supporting), formal analysis (equal), visualization (equal), writing – original draft (equal). **Tong Chen:** data curation (lead), formal analysis (equal), investigation (equal), writing – original draft (equal). **Wei He:** methodology (equal), writing – review and editing (equal). **Zeyao Zhao:** data curation (supporting), investigation (supporting). **Jie Fan:** formal analysis (supporting), software (supporting). **Li Mei:** conceptualization (lead), funding acquisition (lead), methodology (supporting), resources (lead), supervision (lead), writing – review and editing (lead).

## Conflicts of Interest

The authors declare no conflicts of interest.

## Supporting information


**Figure S1.** Residual rates of leaf litter and fine root litter mass, carbon, nitrogen, and phosphorus (Mean ± S.E.) at different decomposition times for different plant functional types (PFTs). The same lowercase denotes non‐significant differences among different decomposition times within each PFT, and the same uppercase denotes non‐significant differences among PFTs at the same decomposition time (*p* < 0.05).

Table S1.
Table S2.
Table S3.
Table S4.

## Data Availability

The data supporting the results are available from the http://datadryad.org/stash/share/pnR3Dhvlv4QADucjGLmWihoXhoE_LeWwo8d9mWKAgjw. https://doi.org/10.5061/dryad.pk0p2ngzb.
