# Supplementary figures and images for: Exploring Litter Decomposition, Nutrient Retention, and Sensitivity to Nitrogen Deposition Among Ancient and Recently Evolved Tree Species
Source: Ecol Evol. 2025 Apr 19;15(4):e71317. doi: 10.1002/ece3.71317 (PMC12008664; doi:10.1002/ece3.71317)

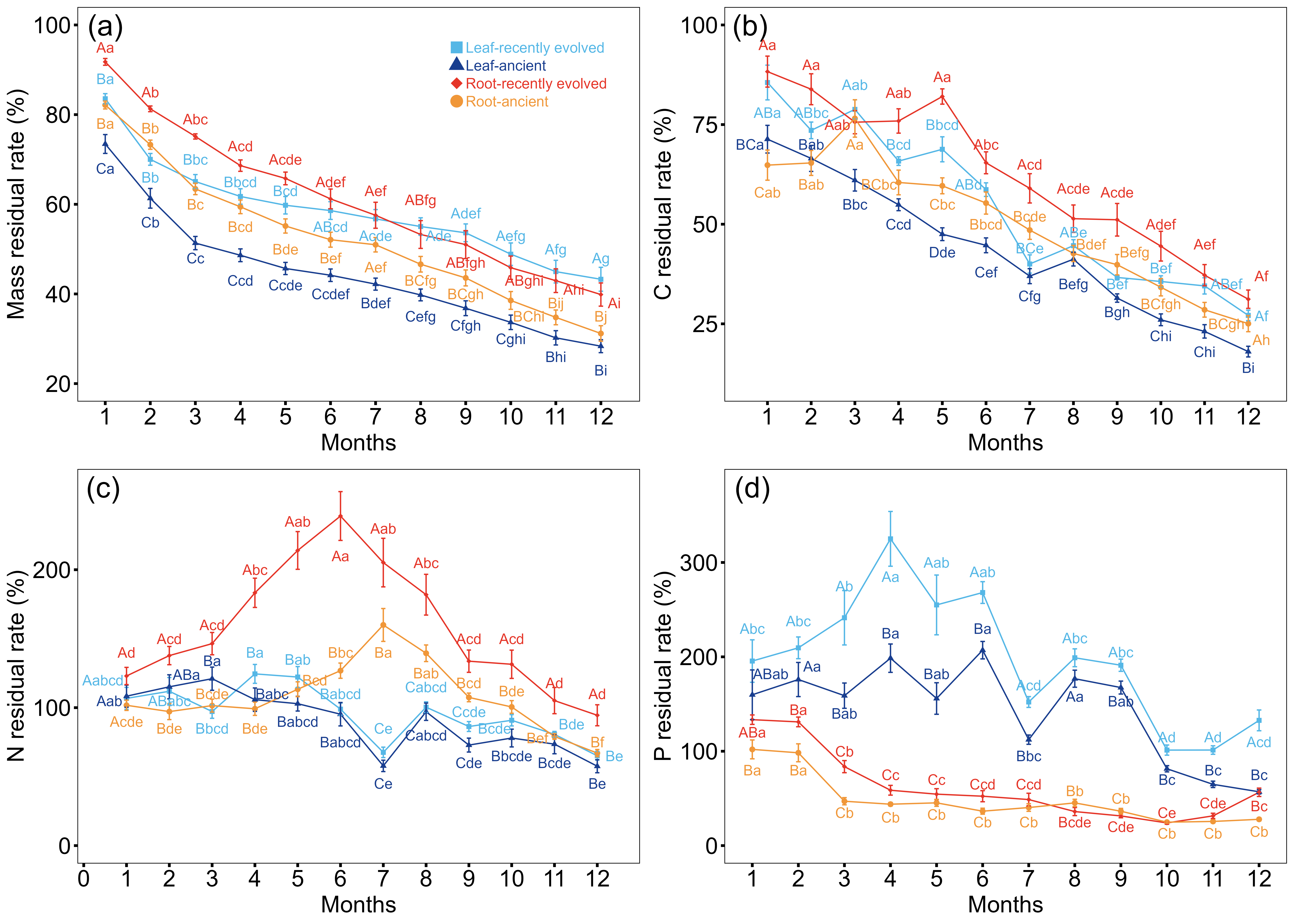

Supplement: Supplementary file 1 — Figure S1. Residual rates of leaf litter and fine root litter mass, carbon, nitrogen, and phosphorus (Mean ± S.E.) at different decomposition times for different plant functional types (PFTs). The same lowercase denotes non‐significant differences among different decomposition times within each PFT, and the same uppercase denotes non‐significant differences among PFTs at the same decomposition time (p < 0.05). [file ECE3-15-e71317-s002.png]
