# Supplementary material for: Exploring Litter Decomposition, Nutrient Retention, and Sensitivity to Nitrogen Deposition Among Ancient and Recently Evolved Tree Species
Source: Ecol Evol. 2025 Apr 19;15(4):e71317. doi: 10.1002/ece3.71317 (PMC12008664; doi:10.1002/ece3.71317)
Supplement: Supplementary file 2 — Table S1. Table S2. Table S3. Table S4. [file ECE3-15-e71317-s001.docx]

**Appendices**

| PFTs | Latin name | Family | Genus | Origin of species | Mycorrhizal type | Coordinates |
| --- | --- | --- | --- | --- | --- | --- |
| Ancient | *Liriodendron chinensis* | Magnoliaceae | Liriodendron | Late Cretaceous | AMF | 30.93N,112.916E |
|  | *Eucommia ulmoides* | Eucommiaceae | Eucommia | Late Cretaceous | ECM | 0.91N,112.909E |
|  | *Liquidambar formosana* | Hamamelidaceae | Liquidambar | Tertiary Paleocene | ECM | 0.91N,112.908E |
| Evolved | *Ilex purpurea* | Aquifoliaceae | Ilex | Eocene tertiary | ECM | 0.95N,112.921E |
|  | *Paulownia tomentosa* | Paulowniaceae | Paulownia | Eocene tertiary | AMF | 30.93N,112.917E |
|  | *Quercus acutissima* | Fagaceae | Quercus | Tertiary Miocene | ECM | 30.92N.112.906E |

**Table S1** The 6 common tree species list and their family names, genus names, functional types, origin of species , and coordinates in Taizi Mountain.

**Table S2** The differences in the first and second axes of PC1 and PC2 of leaf traits and fine root traits from Principal Components Analysis (PCA) between different plant functional types (PFTs) including ancient species and recently evolved species.

| PFTs | Leaf traits | | Root traits | |
| --- | --- | --- | --- | --- |
|  | PC1 (35.7%) | PC2 (31.7%) | PC1 (54.2%) | PC2 (21.1%) |
| Ancient | -1.36±0.26 | 0.432±0.677 | 1.33±0.466 | -0.319±0.538 |
| Evolved | 1.36±0.466 | -0.432±0.247 | -1.33±0.635 | 0.319±0.243 |
| ANOVA | F (1,22) =26.06  *P* < 0.001 | F (1,22) =1.439  *P* = 0.243 | F (1,22) =11.47  *P* < 0.01 | F (1,22) =1.169  *P* = 0.291 |

**Table S3** The differences in initial traits of leaf traits and fine root traits among different plant functional types, including ancient and recently evolved species. The P values in bold denote significant difference among plant functional types at P < 0.05.

| Traits | PFTs | | *t statistic* | *df* | ***P* value** |
| --- | --- | --- | --- | --- | --- |
|  | Ancient | Evolved |  |  |  |
| Leaf C | 414.7±8.31 | 455.767±18.813 | -1.997 | 11 | 0.064 |
| Leaf N | 11.2±0.694 | 11.172±0.387 | 0.036 | 11 | 0.972 |
| Leaf P | 0.34±0.020 | 0.253±0.015 | 3.476 | 11 | **0.002** |
| Leaf C:N | 38.358±2.060 | 41.031±1.597 | -1.026 | 11 | 0.317 |
| Leaf C:P | 1271.035±86.249 | 1822.207±116.044 | -4.040 | 11 | **<0.001** |
| Leaf N:P | 35.519±4.457 | 44.881±1.617 | -1.975 | 11 | 0.069 |
| Leaf Lignin | 16.014±0.811 | 22.497±1.014 | -4.994 | 11 | **<0.001** |
| Leaf Cellulose | 7.750±0.463 | 10.833±0.505 | -4.502 | 11 | **<0.001** |
| Specific leaf area | 20030.54±6934.11 | 11101.91±4101.158 | 1.108 | 8 | 0.288 |
| Leaf thickness | 0.311±0.043 | 0.295±0.044 | 0.269 | 8 | 0.791 |
| Root C | 391.633±7.363 | 399.7±13.960 | -0.511 | 11 | 0.616 |
| Root N | 5.499±0.096 | 3.878±0.214 | 6.922 | 11 | **<0.001** |
| Root P | 0.741±0.078 | 0.559±0.037 | 2.106 | 11 | 0.052 |
| Root C:N | 71.590±2.219 | 108.756±9.967 | -3.640 | 11 | **0.003** |
| Root C:P | 633.538±93.748 | 753.013±57.401 | -1.087 | 11 | 0.291 |
| Root N:P | 8.674±1.125 | 7.074±0.359 | 1.355 | 11 | 0.198 |
| Root Lignin | 16.851±1.368 | 21.036±2.391 | -1.519 | 8 | 0.153 |
| Root Cellulose | 7.334±0.448 | 5.466±0.643 | 2.386 | 8 | **0.031** |
| Root Diameter | 0.810±0.556 | 0.556±0.045 | 2.810 | 8 | **0.015** |
| Specific root length | 15.189±1.770 | 28.226±3.223 | -3.546 | 8 | **0.004** |

**Table S4** The differences in C, N, P, C:N, C:P, N:P, Lignin and Cellulose between leaf and fine root tissues across 6 species. The P values in bold denote significant difference among plant organs at *P* < 0.05.

| Trait | Leaf tissue | Root tissue | *t statistic* | *df* | *P* value |
| --- | --- | --- | --- | --- | --- |
| C | 435.233±10.931 | 395.667±7.764 | 2.284 | 23 | **0.032** |
| N | 11.186±0.389 | 4.689±0.204 | 15.741 | 23 | **<0.001** |
| P | 0.297±0.015 | 0.650±0.046 | -9.814 | 23 | **<0.001** |
| C:N | 39.694±1.305 | 90.173±6.320 | -7.936 | 23 | **<0.001** |
| C:P | 1563.121±93.318 | 693.275±55.179 | 11.263 | 23 | **<0.001** |
| N:P | 40.2±2.516 | 7.874±0.601 | 14.752 | 23 | **<0.001** |
| Lignin | 19.49±1.145 | 18.943±1.429 | 0.552 | 17 | 0.588 |
| Cellulose | 9.444±0.544 | 6.4±0.442 | 3.930 | 17 | **0.001** |
